# Diagnostic and therapeutic value of appendoscope-assisted endoscopic retrograde appendicitis therapy

**DOI:** 10.1055/a-2650-2623

**Published:** 2025-07-29

**Authors:** Pengcheng Liu, Liqiong Wu, Pengju Li, Yu Li, Dianzuo Sun

**Affiliations:** 1 Department of General Surgery, The People's Hospital of Zhuanglang County, Zhuanglang, Gansu, China; 2 Digestive Endoscopy Center, The People's Hospital of Zhuanglang County, Zhuanglang, Gansu, China

**Keywords:** Endoscopy Lower GI Tract, Diagnosis and imaging (inc chromoendoscopy, NBI, iSCAN, FICE, CLE...), Endoscopy Upper GI Tract, Dilation, injection, stenting, GI surgery

## Abstract

**Background and study aims:**

The appendoscope, derived from the peroral digital single-operator cholangioscope, is an endoscopic device enabling direct visualization of the appendix lumen for diagnostic or therapeutic purposes. This study aimed to investigate diagnostic and therapeutic efficacy of appendoscope-assisted endoscopic retrograde appendicitis therapy (ERAT) in patients with appendicitis.

**Patents and methods:**

A total of 131 patients were enrolled in the study, with 125 included in the final analysis. Patient demographics, procedure success, abdominal pain resolution, appendoscope manifestations, treatment strategies, procedure time, duration of antibiotic use, postoperative hospital stay, and comorbidities were recorded. Complications and recurrences were followed up. These variables were subsequently analyzed to evaluate efficacy of appendoscope-assisted ERAT.

**Results:**

The technical success rate of appendoscope-assisted ERAT was 98.5%, and the clinical success rate was 100%. Appendoscope visual manifestations included appendicolith (76.8%, n = 96), stenosis (16.8%, n = 21), foreign body (6.4%, n = 8), mucosal inflammation (13.6%, n = 17), and perforation (3.2%, n = 4), with these findings occurring individually or in combination. Abdominal pain disappeared within 12 hours post-procedure in 79% of patients (n = 99). Average procedure time was 50.3 ± 18.9 minutes. Antibiotic therapy duration was less than 2 days in 39% of patients (n = 49), whereas 19% (n = 24) received no antibiotics. Average postoperative hospital stay was 1.9 ± 1.4 days. Concomitant intestinal lesions were identified in 16 patients (12.8%). Recurrence occurred in 4.8% of patients (n = 6) during a 4- to 16-month follow-up. No complications were recorded.

**Conclusions:**

Appendoscope-assisted ERAT is a feasible, accurate, safe, and effective alternative for diagnosis and treatment of appendicitis.

## Introduction

The etiology of appendicitis is primarily attributed to the appendix’s anatomical structure, most commonly caused by obstruction of the appendiceal lumen due to appendicoliths, tumors, or lymphoid hyperplasia 1 . Current therapeutic approaches for appendicitis include antibiotic therapy and surgical intervention. Numerous studies have demonstrated that irrigation of the appendiceal lumen via endoscopic retrograde appendicitis therapy (ERAT) can alleviate inflammation and prevent disease progression 2 . Although antibiotic therapy offers short-term advantages such as expedited recovery and improved quality-of-life scores compared with surgery, it provides inadequate source control for luminal obstruction and carries significant long-term recurrence risks 3 4 5 6 7 . Although appendectomy remains the established definitive treatment 8 , contemporary understanding rejects the appendix as a vestigial organ 9 . It contains substantial lymphoid tissue 10 and functions as a microbiota reservoir, which restores intestinal flora following dysbiosis or antibiotic disruption 11 . Appendectomy may consequently induce sequelae, with clinical evidence suggesting an increased risk of colorectal cancer, gallstones, and Crohn’s disease 12 13 14 . Furthermore, 10% to 18% of appendectomies are negative resections (histologically normal appendix) and diagnostic advancements have failed to reduce unnecessary procedures 15 16 17 . Consequently, the decision to retain or remove the appendix warrants careful deliberation, and appendectomy as “gold standard” for managing acute appendicitis has been challenged.

ERAT has emerged as an organ-preserving approach for acute uncomplicated appendicitis through fecalith extraction and appendiceal lumen irrigation 2 . Appendoscope-assisted ERAT utilizing a miniaturized endoscopy (“appendoscope”) enables direct visualization of the appendiceal lumen, assessment of mucosal features, fecalith exclusion, lumen irrigation, stent placement assistance, and tissue biopsy acquisition 18 19 . Although prior trials have evaluated appendoscope-assisted ERAT, its application remains limited by small sample sizes and restriction to uncomplicated cases. This study, therefore, investigated the diagnostic value, treatment efficacy, and potential complications of appendoscope-assisted ERAT. The trial design stems from recognition that the appendoscope provides accurate visualization of appendiceal lumen 18 ; accordingly, a classification system based on appendoscope visual manifestations was employed to analyze clinical outcomes and complications.

## Patients and methods

### Study design and protocol

Following institutional review board approval, we retrospectively analyzed patients diagnosed with appendicitis who underwent appendoscope-assisted ERAT at People’s Hospital of Zhuanglang County between July 2023 and August 2024. Inclusion criteria were: 1) typically clinical presentations including right lower quadrant abdominal pain with fever, nausea, or vomiting, plus imaging evidence (computed tomography or ultrasound) indicating appendiceal inflammation (appendix diameter > 6 mm with wall thickening, periappendiceal fat inflammation, and/or periappendicea fluid collection/abscess) 20 ; 2) Alvarado score ≥ 5 21 ; and 3) right lower quadrant pain with radiologically confirmed appendicolith. Exclusion criteria were: 1) failed appendiceal orifice cannulation; 2) concurrent acute abdominal pathologies (cholecystitis, pancreatitis, urinary calculi); 3) suspected neoplasms requiring surgical referral; 4) appendicitis ruled out by colonoscopy; and 5) loss to follow-up.

Given potential risks of ERAT in complicated appendicitis, patients with imaging-confirmed complicated appendicitis were generally recommended for appendectomy unless they expressed strong organ-preservation preferences or had contraindications to surgery. For all enrolled patients, laparoscopic appendectomy was performed promptly if ERAT failed or complications occurred.

Based on previous evidence demonstrating diverse endoscopic manifestations of appendicitis 18 , patients were stratified into prespecified subgroups according to appendoscopic findings: 1) obstructive appendicitis (appendicolith, foreign body, or luminal stenosis without mucosa alterations); and 2) inflammatory appendicitis (mucosa inflammation or perforation, with or without fecalith). The endoscopic diagnostic criterion for appendiceal perforation was direct visualization of periappendiceal fat (with or without fecalith).

### Appendoscope description

The appendoscope used in this study was a single-use video pancreaticobiliary scope (eyeMAX, Micro-Tech (Nanjing) Co., Ltd.; 9F diameter). This disposable digital system features four channels: working, optical fiber, and dual irrigation channels. With a maximum outer diameter of 3 mm, the device is compatible with endoscopes possessing ≥ 3.2-mm biopsy channels. The distal tip provides four-way steering capability, facilitating direct appendiceal orifice cannulation. This enables dynamic, high-definition visualization of luminal structures and contents, allowing precise diagnosis and treatment of inflammation, appendicoliths, stenosis, and neoplasms.

### Appendoscope-assisted ERAT procedure

All patients received intravenous analgesics 30 minutes prior to the procedure and were placed in the left lateral decubitus position. The ERAT procedure was then performed by two operators using the following steps.

For the initial colonoscopy, a colonoscope (CF-H290I, Olympus, Japan) fitted with a transparent cap (Olympus, Japan) at its distal end was advanced to the ileocecal junction. The appendiceal orifice was assessed for signs of inflammation or other abnormalities (the transparent cap helped maintain distance between the lens and mucosa, ensuring complete visualization).

Appendiceal cannulation then was performed. The appendoscope was introduced through the colonoscope biopsy channel. Under direct visualization, the appendiceal lumen was cannulated. In cases of appendiceal strictures, a zebra guidewire (Micro-Tech, China) assisted cannulation. Successful cannulation allowed visual examination of the appendix using the appendoscope.

Next, lavage and stone extraction was performed. The appendiceal lumen was irrigated with 50 to 100 mL of normal saline until fecaliths, pus, or foreign bodies were flushed out and the lumen cleared. For large stones, an extraction basket (Micro-Tech, China) was used for removal.

If applicable, stenosis dilation was performed. For stenotic segments, a guidewire was first passed through the stenosis. The outer sheath of the appendoscope was then advanced over the guidewire to dilate the stenosis for 60 seconds.

Stent placement was performed, if applicable. Patients with a swollen appendiceal orifice, luminal stenosis, appendiceal perforation, or significant pus in the appendiceal cavity underwent placement of a single-sided pigtail plastic stent (5F diameter, 5-cm or 7-cm length, Micro-Tech, China) into the appendiceal orifice under guidewire guidance to facilitate drainage. Patients who received a stent were scheduled for readmission and elective stent removal via colonoscopy 2 to 3 weeks after discharge.

To ensure clear visualization of the appendiceal lumen, continuous suction through the appendoscope channel and lumen irrigation were performed throughout the procedure. Air introduction was avoided to prevent bubble formation, with dimethicone administered as needed.

### Preintervention preparation

All patients underwent bowel preparation with 328.8-g polyethylene glycol (PEG) electrolyte powder dissolved in 2000 mL of water, administered orally 4 to 6 hours preoperatively. Patients with poor tolerance or severe symptoms for PEG electrolyte powder received 500 mL of 20% mannitol solution. Those presenting with fever and leukocytosis received antibiotic therapy (2.0 g ceftriaxone in 100 mL of normal saline) immediately after clinical diagnosis. General anesthesia was available for all patients, but was only used in one pediatric case (4-year-old child).

### Postintervention management

Following appendoscope-assisted ERAT, patients not receiving anesthesia returned to the ward independently and initiated a semi-liquid diet. These patients continued anti-inflammatory medication if abdominal pain persisted, with concurrent hot compresses applied to the right lower quadrant.

Discharge occurred upon complete symptom resolution and return to normal dietary intake. Visual analog scale scores (0 = no pain; 1–3 = mild; 4–6 = moderate; 7–9 = severe; 10 = unbearable pain) were recorded pre- and post-procedure. At 2 to 3 weeks post-ERAT, patients with indwelling stents underwent abdominal x-ray to assess stent position (migrated vs. in situ). Retained stents were removed endoscopically. Follow-up via telephone and/or medical record review continued through the study conclusion, with recurrence defined as return of abdominal pain requiring medical intervention.

Patients in whom ERAT failed or who developed severe adverse events (AEs) (including diffuse peritonitis, septic shock, or worsening signs/symptoms post-procedure) underwent laparoscopic appendectomy by surgeons from the same team.

### Outcomes and data selection

We collected clinical data about the following: sex, age, C-reactive protein (CRP) levels, white blood cell count, neutrophil percentage, duration and severity of abdominal pain, appendoscope manifestations, and colonoscopy-detected intestinal lesions. The primary outcomes were technical success and clinical success. Technical success was defined as successful appendoscope intubation into the appendiceal lumen with advancement to the appendix bottom, accompanied by at least one therapeutic intervention (fecalith extraction, lumen cleansing, stenosis dilation, or stent placement). Clinical success was defined as complete symptom resolution, inflammatory indicators normalization, and restoration of normal dietary intake. Secondary outcomes included postoperative pain relief within 12 hours, ERAT treatment strategies, stent migration rates, readmission for stent removal, procedure time, duration of antibiotic therapy, postoperative hospital stay, complications, and recurrence rates.

### Complications

Complications associated with appendoscope-assisted ERAT included preparatory, intraprocedural, and postprocedural events, such as hemorrhage, perforation, diffuse peritonitis, septic shock, and clinical deterioration.

### Statistical analysis

Statistical analyses were performed using IBM SPSS Statistics version 19.0 (IBM Corp., Armonk, New York, United States). The χ ^2^ test or Fisher’s exact test was used as appropriate. Results are presented as mean ± standard deviation (SD) or number (percentage). The significance level was set at *P* ≤ 0.05.

## Results

### Patient characteristics

**Fig. 1:**
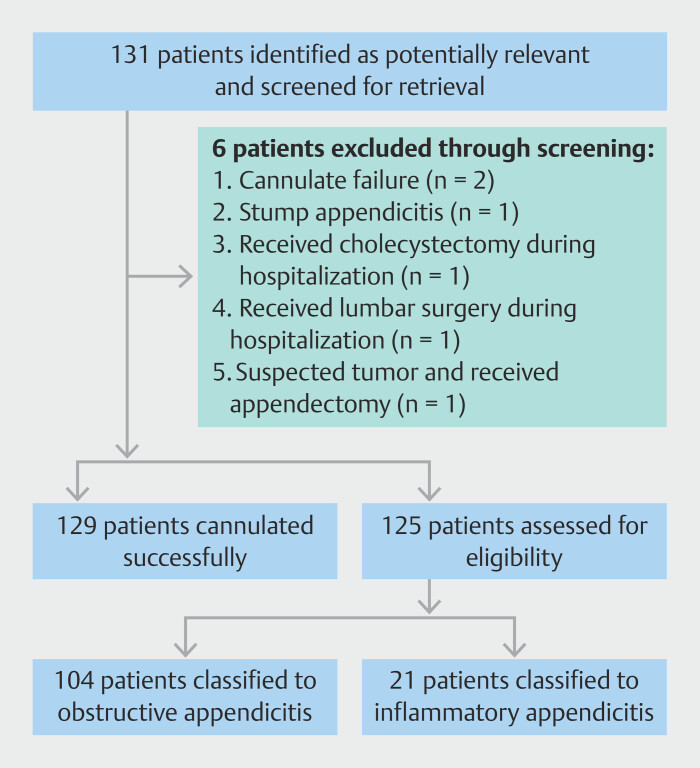
Flow diagram of exclusion criteria and classification.

A total of 131 patients were screened during the retrospective study period ( Fig. 1 ). Of these, 125 patients (58 males, 67 females; age range 4–85 years) met full assessment criteria and were included in the final analysis ( Table 1 ). The cohort included 62 pediatric patients (4–8 years) and one pregnant woman. Based on prespecified subgroups according to appendoscopic findings, patients were classified as having obstructive appendicitis (n = 104) or inflammatory appendicitis (n = 21) ( Table 2 ). Baseline characteristics are detailed in Table 1 .

**Table:** **Table 1** Baseline characteristics and clinical outcomes of all assessed patients.

Patients, n	125
**Sex, n (%)**	
Male	58 (46.4)
Female	67 (53.6)
**Age, median (SD), [range] years**	31.7 (22) [4–85]
Children, n (%)	62 (49.6)
Adult, n (%)	63 (50.4)
CRP ^*^ , n(%)	
0–6 mg/L	107 (85.6)
≥ 6 mg/L	18 (14.4)
** WBC count ^†^ , n(%) **	
> 10×10 ^9^ /L	28 (22.8)
≤ 10×10 ^9^ /L	95 (77.2)
**Neutrophil percentages, n(%)**	
> 75%	33 (26.4)
≤ 75%	92 (73.6)
**The duration of the abdominal pain, n (%)**	
≤ 1 week	48
1week -1 month	18
1 month-6 months	27
≥ 6 months	32
** VAS ^‡^ scores before ERAT, n (%) **	
No pain (0)	0
Mild pain (1–3)	92 (73.6)
Moderate pain (4–6)	33 (26.4)
**VAS scores after 12 hours of ERAT, n (%)**	
No pain (0)	99 (79.2)
Mild pain (1–3)	23 (18.4)
Moderate pain (4–6)	3 (2.4)
**Appendoscope manifestations n (%)**	
Appendicolith	96 (76.8)
Stenosis or adhesion	21 (16.8)
Foreign body	8 (6.4)
Mucosa inflammation (congestion, roughness, swollen, pus or moss)	17(13.6)
Perforation	4 (3.2)
Mucosa inflammation concurrence with appendicolith and/or stenosis	11 (8.8)
**Treatment strategies during ERAT, n (%)**	
Lavage alone	79 (63.2)
Basket extraction	8 (6.4)
Dilatation	21 (16.8)
Stent placement	17 (13.6)
**Stent migrated n(%)**	7 (41.2)
**Readmitted for stent removal n (%)**	10 (58.8)
**Procedure time, mean (SD) [range], minutes**	50.3 (18.9) [30–135]
**Duration of antibiotic therapy n (%)**	
No use	24 (19.2)
≤ 2 days	49 (39.2)
> 2 days	52 (41.6)
**Postoperative hospital stay, mean (SD) [range], days**	1.9 (1.4) [0–8]
≤ 1 day, n (%)	60 (48)
2–3 days, n (%)	51 (40.8)
> 3 days, n (%)	14 (11.2)
**Postoperative complications, n**	0
**Recurrence n (%)**	6 (4.8)
**Comorbidities, n**	
Polyp or adenoma	10
Diverticulitis	5
Pseudomembranous colitis	1
CRP, C-reactive protein; ERAT, endoscopic retrograde appendicitis therapy; SD, standard deviation; VAS, visual analog scale; WBC, white blood cell. *Baseline CRP reference: 0–6 mg/L. ^†^ Baseline leukocytes count reference: 0–10 × 10 ^9^ /L. ^‡^ 0 = no pain; 1–3=mild pain; 4–6 =moderate pain; 7–9 = severe pain; 10 = unbearable pain.	

**Table:** **Table 2** Comparison between obstructive and inflammatory appendicitis groups.

	Obstructive (n = 104)	Inflammatory (n = 21)	*P* value
CRP ^*^ ≥ 6 mg/L, n(%)	8 (8.5)	10 (55.6)	< 0.01
** WBC count ^†^ > 10 × 10 ^9^ /L, ** n (%)	15 (14.3)	13 (61.9)	< 0.01
**Neutrophil percentages > 75%,** n (%)	19 (18.6)	14 (66.7)	< 0.01
** VAS scores ^‡^ ≥ 1 after 12 hours of ERAT ** (n, %)	12 (11.5)	14 (66.7)	< 0.01
**VAS scores ≥ 3 after 12 hours of ERAT** (n, %)	0	3 (14.3)	-
**Stent placement** (n, %)	8 (7.7)	9 (42.9%)	< 0.01
**Procedure time** mean (SD), minutes	46.3 (14.6)	67.8 (28.7)	< 0.01
**Duration of antibiotic therapy** mean (SD), days	2.0 (1.7)	4.7 (3.4)	< 0.01
**Postoperative hospital stay** mean (SD), days	1.6 (1.1)	2.9 (2.1)	< 0.01
**Recurrence** (n, %)	3 (2.9)	3 (14.3)	< 0.01
CRP, C-reactive protein; ERAT, endoscopic retrograde appendicitis therapy; SD, standard deviation; VAS, visual analog scale; WBC, white blood cell. *Baseline CRP reference: 0–6 mg/L. ^†^ Baseline leukocytes count reference: 0–10 × 10 ^9^ /L. ^‡^ 0 = no pain; 1–3 = mild pain; 4–6 = moderate pain; 7–9 = severe pain; 10 = unbearable pain.			

### Primary outcomes

Overall, appendiceal intubation was attempted in 131 patients, with technical success achieved in 129 cases (98.5%, 129/131). Of the two unsuccessful attempts, one patient underwent appendectomy, and the other received antibiotic therapy ( Fig. 1 ). Among the 125 patients who completed the assessment, all recovered successfully, resulting in a clinical success rate of 100%. No patients required conversion to surgery due to complications.

### Abdominal pain

All patients presented with abdominal pain of varying severity prior to the procedure. At 12 hours post-procedure, complete pain relief was achieved in 99 patients (79.2%), whereas 23 (18.4%) reported mild pain and only three (2.4%) experienced moderate pain. Further analysis revealed a statistically significant difference in persistent abdominal pain between groups: 14 patients in the inflammatory appendicitis group (67%, 14/21) reported ongoing pain compared with 12 patients in the obstructive appendicitis group (12%, 12/104) ( *P* < 0.001). Notably, all three patients with moderate pain at 12 hours belonged to the inflammatory group.

### Appendoscope manifestations

**Fig. 2:**
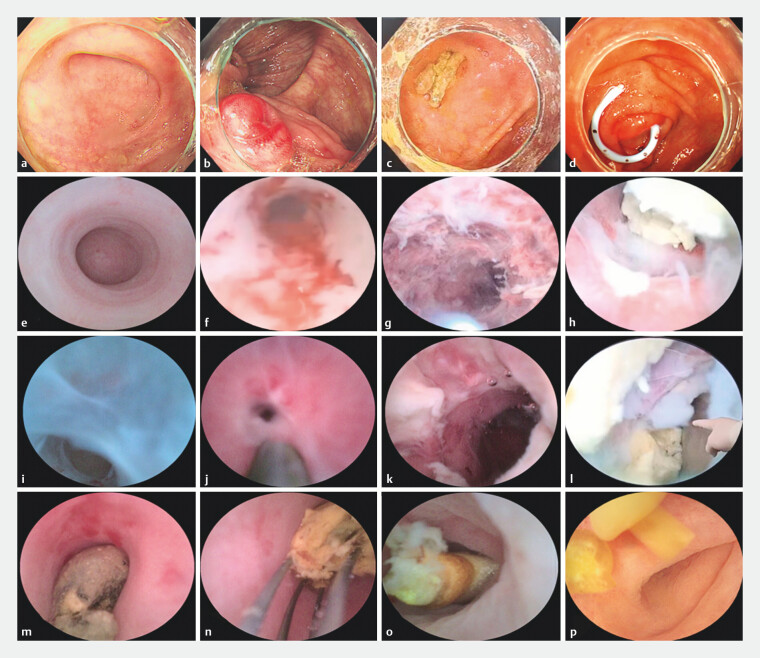
Endoscope manifestations. **a** Normal appendiceal orifice. **b** Colonoscopic view of appendiceal edema. **c** Fecal stone at the appendiceal orifice. **d** Colonoscopic view of a plastic stent at the appendiceal orifice. **e** Appendoscope view of normal appendiceal mucosa and the blind end of the appendix. **f** Appendoscope showing congestion of the appendiceal mucosa. **g** Appendoscope showing rough mucosa in the appendiceal lumen. **h** Appendoscope showing pus and fecal stone in the appendiceal lumen. **i** Appendoscope showing adhesion of the appendiceal mucosa. **j** Appendoscope showing a pinhole-like stenosis in the appendiceal lumen. **k** Appendiceal mucosa after successfully dilation of the stenosis using the anterior end of the appendoscope. **l** Appendoscope showing appendiceal perforation and fecalith. **m** Appendoscope showing a yellow fecal stone embedded in the appendiceal lumen. **n** Appendiceal fecalith removed by an extraction basket under direct vision. **o** Appendoscope showing a foreign body in the appendiceal lumen (grape pip). **p** Colonscopic view of the removed appendiceal foreign body (needle mushroom).

Based on the prespecified subgroups according to appendoscopic findings, 104 patients were classified to obstructive appendicitis group and 21 patients to inflammatory appendicitis group. Appendoscope manifestations included appendicolith in 96 patients (76.8%), stenosis in 21 (16.8%), foreign body in eight (6.4%), mucosa inflammation in 17 (13.6%), perforation in four (3.2%), and mucosa inflammation co-occurring with appendicolith and/or stenosis in 11 patients (8.8%) ( Fig. 2 ).

### Treatment strategies during ERAT

Treatment modalities in this study included lavage alone (63.2%), basket extraction (6.4%; including cases requiring mechanical lithotripsy), dilatation (16.8%), and stent placement (13.6%). Among patients receiving stents, nine were categorized with inflammatory appendicitis and eight with obstructive appendicitis. Ultimately, only 59% (10/17) of stents were electively removed after 2 to 3 weeks; the remaining 41% (7/17) passed spontaneously during defecation.

In the inflammatory appendicitis group, four patients were diagnosed with perforation during the ERAT procedure. These patients had been scheduled for appendectomy regardless of stent drainage, but immediate pain relief followed appendicolith extraction and appendiceal lumen irrigation. Consequently, all four recovered with conservative management during their initial hospitalization. One of these patients developed recurrent abdominal pain 2 months after ERAT and underwent laparoscopic appendectomy; pathological examination confirmed mucinous cystadenoma.

### Procedure time

Procedure time was defined as the interval from colonoscopy initiation to treatment completion. Mean procedure time was 50.3 ± 18.9 minutes. However, mean procedure time for the inflammatory appendicitis group (67.8 ± 28.7 minutes) was significantly longer than that for the obstructive appendicitis group (46.3 ± 14.6 minutes; *P* < 0.001).

### Duration of antibiotic therapy

Duration of antibiotic therapy was defined as the number of days of antibiotic therapy from admission to discharge. Twenty-four patients (19%) received no antibiotics during hospitalization, whereas 49 patients (39%) received antibiotic therapy for fewer than 2 days. Mean duration of antibiotic therapy was significantly longer in the inflammatory appendicitis group (4.7 ± 3.4 days) than in the obstructive appendicitis group (2.0 ± 1.7 days, *P* < 0.001).

### Postoperative hospital stay

Mean postoperative hospital stay was significantly shorter in the obstructive appendicitis group (1.6 ± 1.4 days) than in the inflammatory appendicitis group (2.9 ± 2.1 days; *P* < 0.001). No complications were recorded during or after the procedure.

### Recurrence

The overall recurrence rate during the 4- to 16-month follow-up period was 4.8% (6/125). Among the six patients with recurrence, five had not undergone stent placement during the initial ERAT, and the sixth patient’s stent had discharged spontaneously. Of these six patients, two underwent repeat appendoscope-assisted ERAT with stent placement for adequate dilation/drainage and experienced no further recurrence. Notably, during repeat ERAT, the appendoscope visualized luminal stenosis in both cases. One patient (initially managed with flushing for an appendicolith) experienced recurrent pain after 1 month and underwent laparoscopic appendectomy; pathology confirmed simple appendicitis. One patient (with perforation identified during initial ERAT) developed recurrent pain 2 months post-procedure and underwent laparoscopic appendectomy; pathology indicated mucinous cystadenoma. Two patients experienced mild recurrent abdominal pain, which resolved with antibiotic therapy and local hot compresses, with no further recurrence during follow-up.

Further analysis revealed a significantly lower recurrence rate in the obstructive appendicitis group (2.9%, 3/104) compared with the inflammatory appendicitis group (14.3%, 3/21; *P* < 0.001).

### Comorbidities

Colonoscopy was performed concurrently during the appendoscope-assisted ERAT procedure. Colonoscopic findings of intestinal lesions are summarized in Table 1 and included diverticulitis (n = 5), polyps/adenomas (n = 10), and pseudomembranous colitis (n = 1).

## Discussion

In this study, 125 patients met the inclusion criteria and successfully underwent appendoscope-assisted ERAT. They resumed oral intake immediately post-procedure and achieved full recovery within 8 days. Notably, 48% (60/125) required hospital stays of less than 1 day and nearly 89% (111/125) were discharged within 3 days post-procedure, enabling a prompt return to normal activities. Compared with alternative treatments, ERAT demonstrated faster recovery. The CODA trial 3 , a major randomized study on antibiotic therapy for appendicitis, reported mean missed workdays of 5.26 in the antibiotics group versus 8.73 in the appendectomy group. Further supporting this, Liu’s prospective multicenter randomized clinical trial 22 showed that ERAT resulted in shorter post-procedure hospitalization (3 days vs. 5 days) and earlier dietary resumption (6 hours vs. 48 hours) compared with appendectomy. Our study also highlights a significant advantage of ERAT: markedly reduced antibiotic exposure, with 19% of patients (24/125) requiring no antibiotics and 58% (73/125) receiving antibiotics for ≤ 2 days, substantially less than conservative antibiotic management 3 . In addition, ERAT eliminates the need for general anesthesia and surgical incisions, reducing associated complications and recovery time.

This study summarizes appendoscope manifestations including fecalith, stenosis, foreign body, mucosa inflammation, and perforation. Fecalith was the most prevalent finding (76.8%, 96/125), often amenable to flushing; however, large fecaliths required a stone retrieval basket or mechanical lithotripsy. The utility of electrohydraulic lithotripsy or laser lithotripsy for large stones remains underreported and requires further validation. Stenosis was identified in 21 patients, including three with pinhole-like strictures ( Fig. 2 ). Guidewire-assisted dilation using the appendoscope outer sheath completely relieved abdominal pain in all cases. Eight patients had intra-appendiceal foreign bodies (e.g., fruit pits, seeds, needle mushroom), with immediate resolution of abdominal pain after removal, thereby avoiding unnecessary surgery.

Prior case reports have documented ERAT effectiveness in managing chronic appendicitis, perforated appendicitis, and periappendiceal abscess 23 24 . Notably, four patients in our study with appendiceal perforation identified by appendoscope initially warranted appendectomy, but their abdominal pain resolved immediately after fecalith extraction and lumen irrigation, and they ultimately recovered with conservative management. This outcome may be attributed to the ability of ERAT to evacuate luminal pus, irrigate the appendix with antibiotic solutions, and achieve effective drainage via stent placement. Chronic appendicitis, characterized by mild, persistent abdominal pain, is often underdiagnosed due to atypical symptoms and nonspecific imaging findings 25 . In our cohort, patients with preoperatively suspected chronic appendicitis consistently exhibited appendiceal luminal stenosis, adhesions, or fecaliths on appendoscope visualization. Notably, once stenosis was dilated, adhesions lysed, and fecaliths removed via ERAT, abdominal pain resolved promptly. In conclusion, the appendoscope enables detailed visualization of appendiceal lumen, facilitates etiological treatment, and avoids radiation exposure—advantages particularly significant in our cohort, which included 62 children and one pregnant woman. Identifying the optimal population for appendoscope-assisted ERAT and evaluating long-term therapeutic efficacy remain critical research directions.

Furthermore, patients with inflammatory appendicitis (defined as mucosal inflammation or perforation) exhibited more severe abdominal pain and longer hospital stays compared with those with obstructive appendicitis (defined as fecalith, foreign body, or luminal stenosis without mucosal alterations). Inflammatory cases also demonstrated higher white blood cell counts, CRP levels, and neutrophil percentages than obstructive cases ( Table 2 ). Therefore, our study presents a preliminary visual classification framework for appendoscope-assisted ERAT, which may serve as a practical reference for diagnosing appendicitis severity, guiding intraoperative decisions, and assessing postoperative efficacy. Because no existing reports describe endoscopic grading of appendicitis severity, further research is imperative to validate the methodology proposed herein.

Although no AEs occurred in our study, recurrence remains a concern despite its low incidence. Prior research links appendicoliths with higher recurrence rates in non-surgically managed pediatric appendicitis 26 . The Liu trial reported a 15% (8/52) recurrence rate within 3 years after ERAT 22 , while the Shen study observed a 9% (3/33) recurrence rate with a median 22-month follow-up 27 . Notably, both the Liu and Shen trials utilized radiographic guidance for ERAT, which cannot visualize the appendiceal lumen or ensure deep intubation, thereby risking oversight or underestimation of radiolucent appendicoliths, stenosis, or adhesions, potential contributors to recurrence in traditional ERAT. By contrast, our study employed an appendoscope that allows direct entry into the appendiceal lumen, visualizes its interior, and enables targeted etiology treatment. Here, six patients (4.8%) suffered from recurrence: three (3/104, 2.9%) had obstructive appendicitis and three (3/21, 14.3%) had inflammatory appendicitis. Of these six cases, five did not receive initial appendiceal stenting. When two of these six underwent repeat appendoscope-assisted ERAT, appendiceal stenosis was visualized; symptoms resolved after stenosis dilation and stenting, with no subsequent recurrence. Similarly, Yang et al. reported an 8% (6/76) recurrence rate in patients treated with irrigation alone 28 , but four patients avoided re-recurrence after repeat ERAT with stenting. These findings suggest that inflammation-induced mucosal changes (e.g., stenosis, adhesion) may be primary drivers of recurrence, and that adequate dilation or stent-assisted drainage could be pivotal for reducing recurrence.

Notably, 41% of stents (7/17) dislodged spontaneously within 2 weeks, consistent with prior reports of 31.03% (9/29) spontaneous discharge 27 . Stent migration may correlate with plastic stent diameter/length, appendiceal edema resolution, and intestinal peristalsis. However, asymptomatic spontaneous discharge may be advantageous, sparing patients a second colonoscopy. Future research should establish clear stent placement indications and identify recurrence risk factors.

This study has several limitations. First, appendoscope-assisted ERAT procedures and appendicitis classification rely on operator experience, lacking standardized criteria. Second, the small sample size for perforated appendicitis limits generalizability, necessitating caution until large studies validate ERAT for complicated appendicitis cases. Third, the appendoscope currently serves as an expensive single-use tool (approximately 970 US dollars) and cost-comparison with traditional ERAT is lacking. We posit that development of more affordable disposable or reusable appendoscope-related tools would render this technique more cost-effective and clinically appealing. In addition, incidence of recurrence and long-term complications may have been underestimated due to the relatively short follow-up period. Sustained and extended follow-up is critical to illuminating longer-term outcomes of this approach. Finally, it is important to note that this study utilized a retrospective design and lacked a control group, which may introduce inherent biases and limit the strength of causal inferences drawn from the results.

## Conclusions

In conclusion, this retrospective study demonstrates that appendoscope-assisted ERAT enables accurate etiology diagnosis of appendicitis while serving as a safe and effective treatment modality. Moreover, routine implementation of colonoscopy during ERAT facilitates diagnosis and management of concomitant intestinal lesions. However, endoscopic diagnosis criteria and classification for appendicitis have not yet attracted sufficient academic attention, and long-term efficacy data remain unassessed. Therefore, large-scale international multicenter randomized controlled trials are urgently warranted to further validate the diagnostic and therapeutic value of appendoscope-assisted ERAT.
